# Transcriptomic Analysis of the Cold Resistance Mechanisms During Overwintering in *Apis mellifera*

**DOI:** 10.3390/insects17010059

**Published:** 2026-01-01

**Authors:** Xiaoyin Deng, Yali Du, Jiaxu Wu, Jinming He, Haibin Jiang, Yuling Liu, Qingsheng Niu, Kai Xu

**Affiliations:** 1 Apiculture Science Institute of Jilin Province, Jilin 132108, China; 17836500979@163.com (X.D.); yidingwu02@gmail.com (J.W.); hejinmingmifeng@163.com (J.H.); jhb18047513706@163.com (H.J.); 15843267968@163.com (Y.L.); 13943233663@163.com (Q.N.); 2 Jilin Provincial Key Laboratory of Bee Genetics and Breeding, Jilin 132108, China

**Keywords:** Hunchun bee, overwintering, transcriptome, differentially expressed genes, cold-resistant

## Abstract

Safe overwintering is a challenging issue in rearing management that is inevitably faced by beekeepers in high-latitude regions. Under the combined influence of multiple factors, the overwintering loss rate of Western honey bees has risen continuously, and investigating the molecular mechanisms related to safe overwintering has become key. The Hunchun bee, an *Apis mellifera* ecotype in Jilin Province, China, exhibits strong overwintering ability during an overwintering period of more than five months. To investigate the molecular mechanisms of its cold resistance, we conducted a comparative transcriptomic analysis between the summer breeding period (July) and different overwintering intervals (November, December, January, and February), and then systematically identified key genes and signaling pathways related to cold resistance. The results showed that the highest number of differentially expressed genes (DEGs) was found between December and July. Compared with July, the upregulated genes in Hunchun bee in December were significantly enriched in several pathways, such as ion transport and neuroactive ligand–receptor interactions, and the downregulated genes were significantly enriched in pathways related to fatty acid metabolism, glutathione metabolism, and the peroxisome. Notably, a total of 378 shared DEGs were obtained from the four comparison groups, and several candidate cold-resistant gene families, such as AFPs, HSPs, C_2_H_2_-ZFPs, STKs, and LRRCs, were identified among the shared DEGs of the winter season. Additionally, 749 shared DEGs related to protein modification and metabolic process regulation were identified between the four successive overwintering intervals. Four shared genes, including sensory neuron membrane protein 1 (*SNMP1*), were revealed by pairwise comparison of the four intervals. The above results collectively indicate that the Hunchun bee attenuates winter-induced stress responses during the overwintering process by maintaining osmotic pressure balance, reducing fatty acid metabolism, increasing antioxidant capacity, and synthesizing cold-resistant macromolecular proteins. It was also found that chemical signal perception may serve a role in maintaining the stability of the overwintering bee colony. The key genes and pathways related to cold resistance identified in this study not only provide a basis for explaining the overwintering molecular mechanism for *Apis mellifera* of Hunchun bee but also offer key data to improve overwintering management strategies for Western honey bees.

## Simple Summary

The Hunchun bee is an ecotype of *Apis mellifera*, which is mainly distributed in Hunchun city, Jilin Province, China. It possesses excellent cold resistance, but the molecular mechanisms of its safe overwintering remain unclear. We analyzed the gene expression levels of Hunchun bees during summer breeding and four overwintering intervals, and we found that they mitigated low-temperature-induced stress during winter by maintaining osmotic pressure balance, reducing fatty acid metabolism, and increasing antioxidant capacity. In addition, their resistance to low temperatures was enhanced through the regulation of genes encoding cold-resistant macromolecular proteins, such as antifreeze proteins (AFPs), C_2_H_2_ zinc finger proteins (C_2_H_2_-ZFPs), Heat Shock Proteins (HSPs), Serine/Threonine Kinase Proteins (STKs), and leucine-rich repeat-containing proteins (LRRCs). This study provides a novel approach to investigating the molecular mechanism of overwintering in the Western honey bee.

## 1. Introduction

Honey bees are the most widely used pollinating insects in modern agriculture. Besides providing human beings with bee products of rich nutritional value, they also play an important role in improving crop yield and maintaining ecological security and biodiversity [1]. For intensively grown crops that bloom in spring, such as almonds [2], apples [3] and cherries [4], Western honey bees are the most preferred pollinating insects due to their large colonies and ease of transportation. The survival and loss rates of honey bees during the overwintering period are the most important factors influencing colony development, pollination efficiency, and the feeding cost of pollinator colonies in early spring [5]. However, under the influence of a variety of factors, such as *Varroa mite* parasitism [6], bee disease epidemics [7], extreme weather events [8], and improper feed management [9], the colony loss rate of Western honey bees during the overwintering period has gradually increased worldwide in recent years. In high-latitude regions, their overwintering losses may reach 30–50% [10], which considerably increases the cost of rearing bee colonies used for early spring pollination [11]. Therefore, improving overwintering safety in Western honey bees is a key challenge currently faced by beekeeping industries worldwide.

Winter is one of the most important abiotic factors affecting the growth, development and geographical distribution of insects. Insects can be classified into freeze-tolerant and freeze-intolerant species [12], and studies have shown that freeze resistance is jointly determined by genotype and environmental factors in both types. Current research on insect overwintering has mainly focused on changes in physiological activities during cold acclimation and diapause [13]. Cold acclimation mainly involves the transcriptional regulation of genes related to the synthesis of small-molecule cryoprotectants such as HSPs and glycerol, ion balance maintenance, and the cytoskeletal structure [14,15]. This prevents the formation of ice crystals in the body and thus improves the chances of insect survival in cold environments [16]. Diapause involves physiological activities such as the expression of circadian clock genes [17], insulin signaling [18], and lipid metabolism [19]. Metabolic inhibition during diapause aids insects in conserving energy and resisting temperature stress. Actually, comparing the gene expression levels in insects between different seasons is a major method for exploring cold-resistant candidate genes and related pathways [20,21].

An analysis of key genes and signaling pathways during the overwintering period is key to improving cold resistance in Western honey bees. Honey bees adapt to low-temperature environments in winter by forming tight clusters to maintain hive temperature, relying on honey and pollen reserves as energy sources, generating heat through the vibration of thoracic flight muscles in worker bees, and making physiological adjustments such as lowering their metabolic rate and increasing their fat body reserves to ensure the successful overwintering of colonies [22]. Research on cold resistance in honey bees has made significant progress in recent years, with studies mainly focusing on individual physiological mechanisms, their colony regulation ability, and differences in cold resistance among different species [23,24]. *Apis cerana cerana* enhances freeze resistance through differential expression of AFP genes and reduces the risk of frost damage in tissues by inhibiting ice crystal formation [25]. During the overwintering period, *Apis mellifera ligustica* regulates the glutamine–glycerol metabolism pathway to increase their glycerol content, which lowers the freezing point and glycogenolysis [21,26]. This contributes to the maintenance of blood glucose levels, thereby providing protection against low temperatures and maintaining energy homeostasis [21]. Cold resistance-related genes, such as HSP genes and cold shock protein genes, have been identified in *Apis cerana cerana* from the Changbai Mountains. Such genes influence fat body expansion and insulin signaling pathway inhibition, with cold hardiness genes also associated with the epigenetic regulation of energy storage in the fat body [27]. Exposure to low temperatures during the pupal stage can suppress both the glutathione metabolism and peroxisome pathways, resulting in depleted antioxidant capacity and ultimately inducing neural damage [24]. However, up to now, there have been no reports on the molecular mechanisms of safe overwintering focusing on overwintering Western bees.

The Hunchun bee, an *Apis mellifera* ecotype to Jilin Province, China, possesses exceptionally strong cold resistance and high honey production performance [28]. During the overwintering period, which lasts five to six months, the overwintering colony loss rate of Hunchun bee is 15–17%, which is significantly lower than that of *Apis Mellifera ligustica* in the same region [29]. Our previous study showed that the levels of vitellogenin and juvenile hormones and the expression of related genes in Hunchun bee changed significantly between summer and different overwintering intervals, suggesting that a series of physiological changes occur when Hunchun bee resists low temperatures [28]. In the present study, we performed a transcriptomic analysis of the gene expression of Hunchun bee during the summer breeding period and four overwintering intervals, with the aim of identifying cold-resistant genes and signaling pathways related to overwintering. We hope that our results can provide data support for elucidating the molecular mechanisms of the high cold resistance in the Hunchun bee.

## 2. Materials and Methods

### 2.1. Sample Preparation

All Hunchun bee samples used in this study were obtained from conserved populations kept in the National Bee Gene Bank of China (Jilin, China). Around 1920, Lu Yongjun and others from Hunchun City, Jilin Province, brought back 5 to 10 colonies of Western honeybees with black coloration from Russia, and began to raise these bees in Hunchun City. After over a hundred years of long-term adaptation to the ecological conditions of Hunchun City and artificial selection by local beekeepers, these black bees have evolved into a distinct ecological variant of Western honeybee *Apis mellifera* [29]. On 1 May 2022, three healthy Hunchun bee colonies, each with a colony strength of five combs and a normal laying queen, were selected as experimental colonies. Subsequently, the same methods were adopted for normal rearing in Fengman District, Jilin City, Jilin Province (longitude 126°67′ E, latitude 43°72′ N). Samples collected during different intervals all originated from these three experimental colonies. A total of 30 worker bees (21-day-old after emergence) were collected from each colony at 10:00–10:30 a.m. on 25 July 2022 to serve as samples for the summer breeding period. The rearing of overwintering bees started on 20 August and concluded on 10 September. Tested colonies were transferred to the overwintering room on 20 November 2022, and then moved out of the room on 10 March 2023. Throughout the overwintering period, the colonies were kept in a state of darkness and quietness, and a temperature range of −2–2 °C was maintained consistently. On 25 November, 25 December, 25 January, and 25 February 2023, 30 worker bees were collected from the outside of each colony at 10:00–10:30 a.m. to serve as the overwintering samples. The samples of each period were rapidly subjected to intestinal tract removal, placed in liquid nitrogen, and subsequently stored at −80 °C.

### 2.2. RNA Sequencing and Analysis

A total of 15 biological samples from Hunchun bee, encompassing five experimental groups with three biological replicates each (*n* = 3), were used for transcriptome sequencing. Total RNA was extracted from each sample. RNA integrity and purity were assessed using agarose gel electrophoresis, a NanoPhotometer^®^ spectrophotometer (IMPLEN, Westlake Village, CA, USA), and an RNA Nano 6000 Assay Kit (Agilent Technologies, Santa Clara, CA, USA) on the Agilent Bioanalyzer 2100 system (Agilent Technologies, Santa Clara, CA, USA). Only high-quality RNA samples (RNA Integrity Number, RIN > 7.0) were used for library construction. Sequencing libraries were prepared with the NEBNext^®^ Ultra™ RNA Library Prep Kit from Illumina^®^ (NEB, Ipswich, MA, USA) according to the manufacturer’s instructions. Briefly, mRNA was enriched using oligo(dT) beads and fragmented. First-strand and second-strand cDNAs were synthesized sequentially. Then, the cDNA fragments were end-repaired, adenylated, and ligated with Illumina adapters. After size selection and PCR amplification, the final libraries were quantified for quality and concentration using the Agilent Bioanalyzer 2100 system. The qualified libraries were then sequenced on an Illumina NovaSeq 6000 platform (Illumina, San Diego, CA, USA) at Novogene Co., Ltd. (Beijing, China) to generate 150 bp paired-end reads.

### 2.3. Bioinformatic Data Processing

Raw sequencing data in FASTQ format were quality-controlled using fastp v0.23.4 to remove adapter sequences, poly-N sequences, and low-quality bases, yielding clean reads. The clean reads were then aligned to the reference genome (NCBI Assembly: GCF_003254395.2, Amel_HAv3.1) using an appropriate aligner. Based on the mapping results, transcript assembly and gene expression quantification were performed, with expression levels estimated using the FPKM (Fragments Per Kilobase of transcript per Million mapped reads) metric. Differentially expressed genes (DEGs) were identified by adjusted *p*-value (padj) < 0.05 and |log_2_(fold change)| ≥ 1 using the DESeq2 package in R 4.3.3 software. The shared gene set of the overwintering period was defined as the intersection of DEGs from all six possible pairwise comparisons among the four overwintering intervals. Gene Ontology (GO) and Kyoto Encyclopedia of Genes and Genomes (KEGG) pathway enrichment analyses of DEGs were performed using the clusterProfiler package in R software. The results were consolidated and comprehensively analyzed to elucidate their biological implications.

### 2.4. Enzyme Activity Assays

The activities of four antioxidant enzymes from Hunchun bee, including glutathione (GSH), catalase (CAT), superoxide dismutase (SOD), and peroxidase (POD), were measured over five periods. All assays were performed using commercial reagent kits purchased from the Nanjing Jiancheng Bioengineering Institute (Nanjing, China), strictly following the manufacturer’s protocols. Briefly, bee tissues were homogenized in ice-cold physiological saline, and the resulting supernatants were collected after centrifugation for subsequent enzymatic reactions. The absorbance changes associated with each specific reaction were monitored using a spectrophotometer. The GSH content was determined based on the reaction with DTNB; CAT activity was assessed via the hydrogen peroxide decomposition rate; SOD activity was measured via its ability to inhibit hydroxylamine oxidation; and POD activity was evaluated using the guaiacol method. Enzyme activities were calculated and expressed as units per gram of tissue protein or fresh weight, as defined by the respective kits.

### 2.5. RT-qPCR Validation

To validate the RNA sequencing data, quantitative real-time PCR (RT-qPCR) was performed. Total RNA was reverse-transcribed into first-strand cDNA using a PrimeScript RT reagent kit (TaKaRa, Dalian, China). Gene-specific primers were designed using the Primer Premier software 5 and synthesized by Sangon Biotech (Shanghai, China). The RT-qPCR reactions were carried out in triplicate for each sample using a SYBR Green Premix Pro Taq HS qPCR kit (Guangzhou Haobio Biotechnology Co., Ltd., Guangzhou, China) on 7500 Real-Time PCR System (Thermo Fisher Scientific, Waltham, MA, USA). The thermal cycling conditions were as follows: initial denaturation at 95 °C for 30 s, followed by 40 cycles of 95 °C for 5 s and 60 °C for 30 s. A total of 12 genes were identified using the fluorescence quantitative test. The primer sequences are shown in Table S1.

### 2.6. Data Analysis

The relative expression levels of the target genes were calculated using the 2^−ΔΔCt^ method, with the actin gene serving as an internal reference for normalization. Data are presented as the mean ± standard error (SE) of three independent biological replicates, each measured in technical triplicate. In correlation analysis, RNA-seq fold changes were expressed as Log_2_ (Fold change), while RT-qPCR fold changes were expressed as −ΔΔCt. The statistical significance of differences between groups was determined using a one-way analysis of variance (ANOVA), followed by Tukey’s post hoc test, with a *p*-value of less than 0.05 considered statistically significant. Significance levels are denoted by different letters in the figures.

## 3. Results

### 3.1. Comparison of Sequencing Data with the Reference Genome

**Figure 1:**
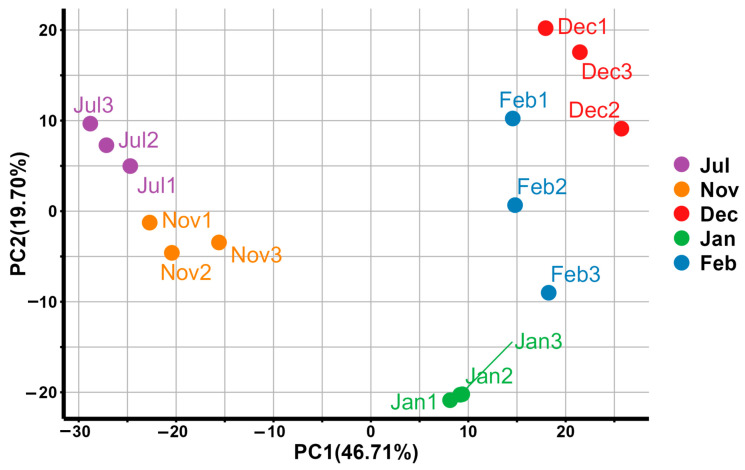
Intra-group and inter-group principal component analysis plot.

A total of 15 RNA-seq libraries from Hunchun bee were constructed in this experiment, which included the data from the following five experimental groups: the Jul group, the Nov group, the Dec group, the Jan group, and the Feb group. The results of the principal component analysis indicate significant differences between groups and minimal variation within groups (Figure 1). After quality control, 40.92–56.86 M raw reads were obtained for each of the 15 RNA-seq libraries, of which 40.25–55.80 M clean reads were obtained after filtering. Q20 and Q30 exceeded 97.61% and 93.17%, respectively. A comparison with the reference genome revealed that the average mapping rate between the various libraries and the reference genome was 92.30%, indicating that the RNA-seq sequencing data of the study met the quality requirements (Table S2).

### 3.2. Differences in Gene Expression Between Summer and Winter

**Figure 2:**
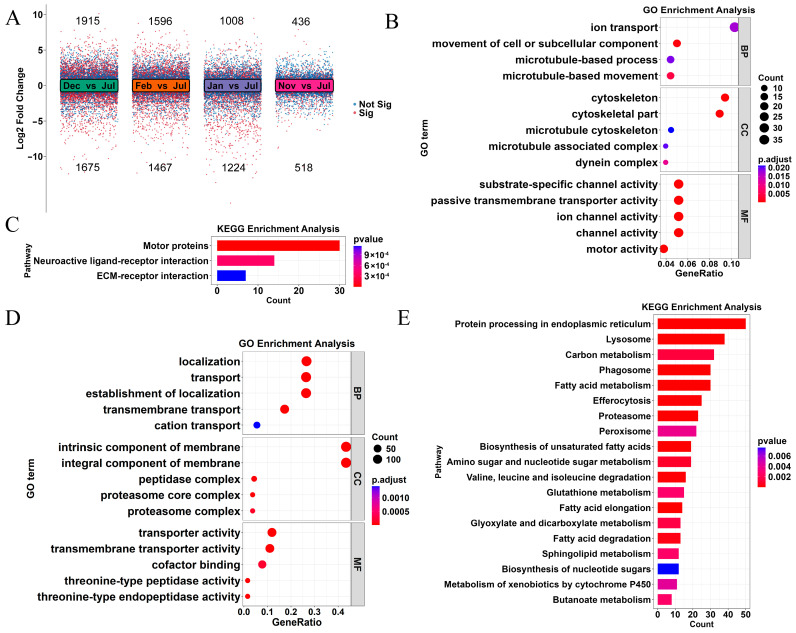
Gene function enrichment analysis of DEGs in the Jul vs. Dec comparison. (**A**) Volcano plot and number of DEGs between the summer breeding period and the four overwintering intervals. The number above the image indicates the number of upregulated genes, while the number below indicates the number of downregulated genes.; (**B**) enriched GO pathways of significantly upregulated DEGs; (**C**) enriched KEGG pathways of significantly upregulated DEGs; (**D**) enriched GO pathways of significantly downregulated DEGs; and (**E**) enriched KEGG pathways of significantly downregulated DEGs.

We first determined the differences in gene expression between the summer breeding period and the four overwintering intervals of Hunchun bee. The results indicated the presence of 954, 3590, 2232, and 3063 DEGs between July and November, December, January, and February, respectively (Figure 2A). Therefore, the Dec vs. Jul comparison, which had the highest number of DEGs, was selected to identify molecular pathway differences in Hunchun bee between winter and summer (Table S3).

Compared with Jul, Dec exhibited 1915 upregulated genes and 1675 downregulated genes. Gene Ontology (GO) enrichment analysis of the 1915 upregulated DEGs indicated that 14 pathways were significantly enriched (*p* < 0.05) (Figure 2B). These included biological process (BP) pathways involved in cell movement or the subcellular component, microtubule-based movement, and ion transport; cellular component (CC) pathways related to the cytoskeleton, the dynein complex, and the microtubule-associated complex; and molecular function (MF) pathways associated with ion channel activity, channel activity, and motor activity. Three significantly enriched pathways were identified based on the Kyoto Encyclopedia of Genes and Genomes (KEGG) enrichment analysis (Figure 2C), namely the motor protein, neuroactive ligand–receptor interaction, and ECM–receptor interaction pathways. GO enrichment analysis of the 1675 downregulated differential genes revealed 15 significantly enriched pathways (*p* < 0.05) (Figure 2D). These included BP pathways involved in transmembrane transport, localization, and cation transport; CC pathways involving the proteasome core complex; and MF pathways associated with transmembrane transporter activity, threonine-type peptidase activity, and cofactor binding. A total of 19 significantly enriched pathways were identified from the KEGG enrichment analysis (Figure 2E), including those related to fatty acid metabolism; the proteasome; valine, leucine, and isoleucine degradation; amino sugar and nucleotide sugar metabolism; carbon and glutathione metabolism; and xenobiotic metabolism by cytochrome P450.

### 3.3. Shared DEGs Between Four Overwintering Intervals and the Summer Breeding Period

Combined analysis of DEGs of the four overwintering intervals with July revealed the existence of 378 shared DEGs, of which 176 were upregulated, while 202 were downregulated (Table S4).

GO enrichment analysis of the 176 upregulated genes indicated an absence of enriched BP and CC pathways, with only five MF pathways (e.g., serine-type peptidase activity) showing significant enrichment (Table S5). From the analysis of 20 BP pathways that were most enriched for DEGs (Table S5A), pathways related to localization, transport, responses to stimulus, the macromolecule biosynthetic process, signal transduction, and the protein modification process were identified. Among the top 10 enriched KEGG pathways with the highest number of DEGs, the MAPK signaling pathway, neuroactive ligand–receptor interactions, the Wnt signaling pathway, cofactor biosynthesis, and the Notch signaling pathway were identified (Table S5C). Subsequently, GO enrichment analysis was performed on the 202 downregulated genes, and the following pathways were identified from the 20 BP pathways most enriched for DEGs: the localization, oxidation–reduction process, ion transport, phosphate-containing compound metabolic process, response to stimulus, proteolysis, and macromolecule biosynthetic process pathways (Table S5B). Among the top 10 enriched KEGG pathways with the highest number of DEGs, those related to carbon metabolism, amino sugar and nucleotide sugar metabolism, fatty acid metabolism, the peroxisome, and oxidative phosphorylation were identified (Table S5D).

### 3.4. Identifying DEGs Related to Cold Resistance

**Figure 3:**
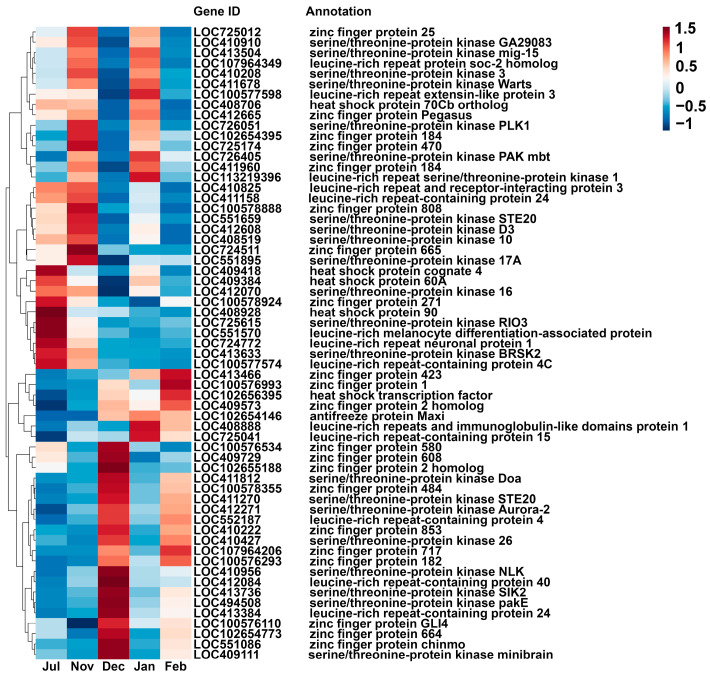
The heat map of DEGs related to cold resistance was identified in different groups. The abscissa represents different groups, and the ordinate is the differentially expressed genes.

Based on the existing literature, we identified DEGs belonging to four candidate cold-resistant gene families, namely AFPs, C_2_H_2_-ZFPs, STKs, and LRRCs (Figure 3). A total of 57 DEGs including one AFP, six HSPs, 22 C_2_H_2_-ZFPs, 18 STKs, and 10 LRRCs were identified in Jul vs. Dec. Among the shared DEGs between the summer breeding period and four overwintering intervals, one HSP, one C_2_H_2_-ZFP, two STKs, and seven LRRCs were identified.

### 3.5. Analysis of Shared DEGs Across Successive Overwintering Intervals

**Figure 4:**
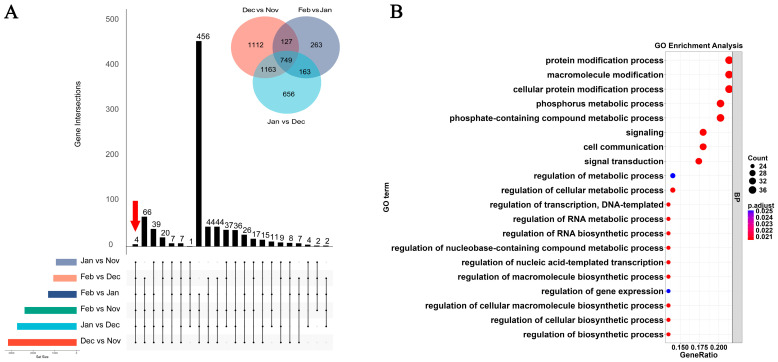
Identified and GO enrichment analysis of shared DEGs across successive overwintering intervals. (**A**) The upset diagram figure of six comparison groups. The first column represents the common differentially expressed genes between the six groups during the overwintering period. The upper right corner of the figure represents the common differentially expressed genes in Dec vs. Nov, Jan vs. Dec, and Feb vs. Jan; (**B**) The significant enrichment GO terms of shared DEGs across successive overwintering intervals.

Based on the combined analysis of DEGs for the Dec vs. Nov, Jan vs. Dec, and Feb vs. Jan comparisons, we identified 749 DEGs shared between successive overwintering intervals (Figure 4A). However, no shared upregulated genes or downregulated genes were identified. From GO enrichment analysis of the above genes, a total of 20 significantly enriched BP pathways were identified (*p* < 0.05), including those related to the protein modification process, the phosphate-containing compound metabolic process, the regulation of the nucleobase-containing compound metabolic process, signal transduction, the regulation of the macromolecule biosynthetic process, gene expression regulation, and regulation of metabolic processes (Figure 4B). Significantly enriched CC and MF pathways were not found. The results of the KEGG enrichment analysis revealed the presence of only one significantly enriched pathway, namely the efferocytosis pathway.

### 3.6. Shared Gene Set of the Overwintering Period

Based on the pairwise comparison and analysis of DEGs of the Hunchun bee for the four overwintering intervals, four shared genes, including *SNMP1* (LOC413995), polypeptide N-acetylgalactosaminyltransferase (LOC410470), the nose resistant to fluoxetine protein (LOC411564), and ABC transporter G family member 20 (LOC724865), were identified.

### 3.7. Antioxidant Capacity Hunchun Bee

**Figure 5:**
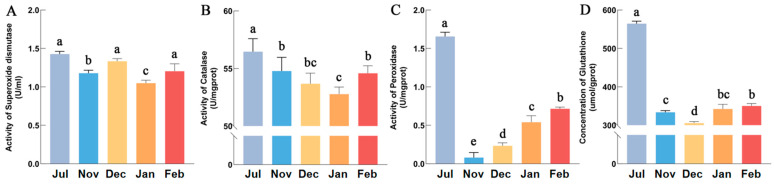
The activities of SOD (**A**), CAT (**B**), POD (**C**) and the concentration of GSH (**D**) in different groups. Lowercase letters above each bar represent significant differences (*p* < 0.05) (Tukey’s honestly significant difference test).

Through functional annotation of DEGs, the oxidative-reduction pathway was identified as significant in this study. Thus, we conducted an enzyme activity assay to validate this result and explore the correlation between the oxidative-reduction process and cold resistance in Hunchun bees. Compared to summer, the activities of the antioxidant enzymes SOD, CAT, POD and the concentration of GSH decreased during overwintering period. As winter progresses, the changing trends of four antioxidants in Hunchun bee exhibit differences. The SOD activities showed a fluctuating trend of first rising, then falling, and then rising again (Figure 5A). The CAT activities and the concentration of GSH showed earlier decrease and later increase trend (Figure 5B,D). The POD activity continues to increase during the whole overwintering period (Figure 5C).

### 3.8. Verification of Transcriptome Gene

**Figure 6:**
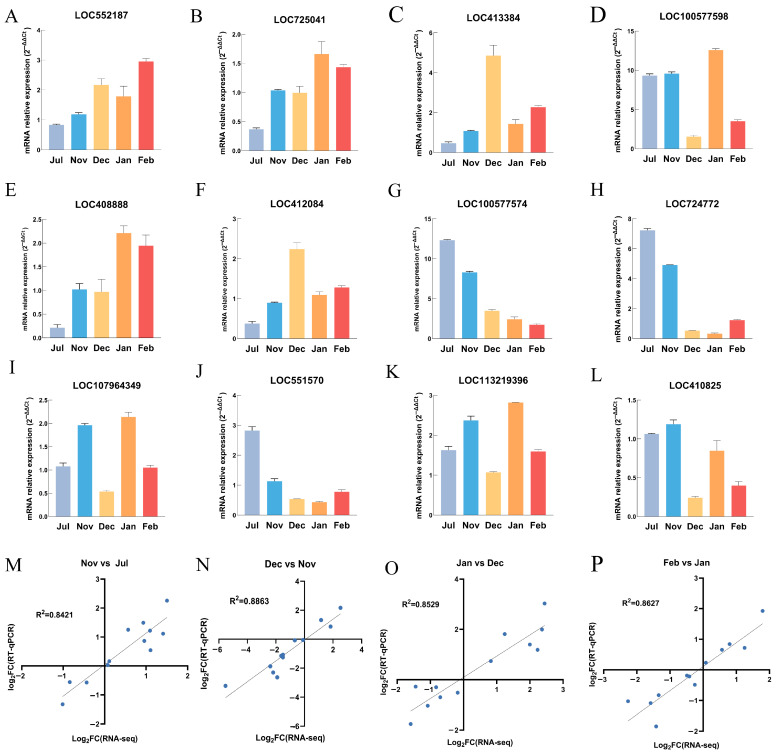
Verification of transcriptome gene. (**A**–**L**) The 12 LRRC genes, including LOC552187, LOC725041, LOC413384, LOC100577598, LOC408888, LOC412084, LOC100577574, LOC724772, LOC107964349, LOC551570, LOC113219396 and LOC410825 to perform RT-qPCR detection; (**M**–**P**) Correlation analysis between RT-qPCR and RNA-seq.

LRRC family genes have been extensively investigated in the context of plant cold resistance mechanisms but remain largely unexplored as candidate genes for insect cold resistance. To address this gap and bolster the credibility of their association with cold resistance, we randomly selected 12 LRRC genes, including LOC552187, LOC725041, LOC413384, LOC100577598, LOC408888, LOC412084, LOC100577574, LOC724772, LOC107964349, LOC551570, LOC113219396 and LOC410825 to verify the accuracy of the RNA-seq data. The RT-qPCR data were significantly correlated with the RNA-seq results, with correlation coefficients of 0.842 (Nov vs. Jul), 0.8863 (Dec vs. Nov), 0.8529 (Jan vs. Dec), and 0.8627 (Feb vs. Jan), thus confirming the validity of the transcriptome dataset (Figure 6M–P).

## 4. Discussion

### 4.1. Osmoregulatory Capacity Is Critical for Overwintering in Hunchun Bee

Most insect species of the orders Diptera, Hemiptera, Lepidoptera, and Orthoptera in tropical and temperate zones are classified as chill-susceptible based on their cold resistance. The cold resistance of such species depends on their ability to maintain physiological homeostasis at low temperatures [30]. With a decrease in temperature, the active transport of solutes in insect cells and tissues becomes significantly retarded. When the critical temperature is reached, organism homeostasis becomes imbalanced. This leads to a continuous reduction in the water and Na^+^ contents of the hemolymph and a continuous rise in K^+^ concentration, thereby triggering hyperkalemia onset [31]. Hyperkalemia triggers cold-induced cellular depolarization, which is exacerbated by the loss of electrogenic potential, ultimately leading to the loss of neuronal and muscular excitability, inducing chill coma [32]. This cold depolarization may also activate voltage-dependent Ca^2+^ channels and promote Ca^2+^-activated apoptosis and necrosis [33]. Cold-tolerant insects also adjust their osmoregulatory capacity to preserve extracellular homeostasis in the cold by reducing leakages and/or inducing changes to active transport systems to counteract the buildup of hemolymph. In addition, certain insects also use ion cotransporter and neuroendocrine regulators to control the transport rates of osmoregulatory organs and therefore cold resistance [34]. Unlike the solitary insects mentioned above, honey bees belong to the order Hymenoptera. Temperature is a key abiotic factor directly shaping the geographical distribution and population dispersal of honey bees [22]. The cold-limited geographic range of Africanized honey bees underscores the importance of thermal adaptation within *Apis mellifera* [35]. During the winter season, worker bees resist cold temperatures by forming clusters and generating heat through the consumption of food reserves. Therefore, cold-induced coma will not occur in worker bees under safe colony overwintering conditions. In the present study, the DEGs of Dec vs. Jul, Dec vs. Nov, and between the early overwintering period and different overwintering intervals were significantly enriched (*p* < 0.05) in molecular pathways related to ion transport, membrane components, transmembrane transporter protein activity, neuroactive ligand–receptor interactions, motor proteins, and phospholipid metabolism. This suggests that honey bees maintain physiological homeostasis by adjusting their osmoregulatory capacity to avoid cold-induced coma, which is similar to the strategy adopted by insects of other orders. However, the specific ion transport mode of honey bees in low-temperature environments remains to be elucidated.

### 4.2. Antioxidant Stress Is Critical for Overwintering in Hunchun Bee

Oxidative stress is a major lethal stress response in animals faced with various environmental stresses [36]. During oxidative stress, the balance between reactive oxygen species (ROS) production and the organism’s scavenging capacity is disrupted, leading to a rapid increase in ROS levels. This impairs the oxidant scavenging system, thus causing oxidative damage to proteins, lipids, and nucleic acids. Consequently, cellular and organ functions are disrupted, and death may occur in severe cases [37]. In insects, oxidative stress can be induced by an increase in ROS levels, which occurs due to exposure to herbicides and pesticides, ultraviolet light, and low temperatures. The upregulation of anti-oxidative stress-related genes, such as the glutathione (GSH), superoxide dismutase (SOD), catalase (CAT), and peroxidase (POX) transcription factors, in the organism can inhibit oxidative damage to biomacromolecules, increase resistance to oxidative stress, and prolong the organism’s lifespan [38,39]. In particular, SOD catalyzes the cleavage of superoxide radicals to oxygen and H_2_O_2_, while both CAT and POX catalyze H_2_O_2_ breakdown to oxygen and water, and GSH eliminates lipid peroxidation products or hydroperoxides in cells. In a recent study, Western honey bee pupae were exposed to a low temperature of 20 °C for 24 h or 48 h, and it was found that GSH metabolism and peroxisome function were inhibited under low-temperature stress [24]. In this study, we found that downregulated DEGs common to the four overwintering intervals vs. Jul were significantly enriched in redox-related signaling pathways. Further enzyme activity assays results showed that the activities of SOD, CAT, and POD and the concentration of GSH were downregulated during the overwintering intervals compared to summer, it results suggested that low temperatures in winter suppressed activity of antioxidant enzymes, inducing oxidative stress. Surprisingly, we found activities of SOD, CAT, and POD and the concentration of GSH were higher in the middle and later stages of overwintering, this implies that improving the antioxidant capacity is key to enhancing the cold resistance of Hunchun bee.

### 4.3. Fatty Acid Metabolism Degradation Is an Overwintering Strategy of Hunchun Bee

For most insects, fat accumulation before overwintering and fat consumption for energy provision during overwintering are essential for safe overwintering [24]. Fat serves as the main tissue regulating the cold stress response in insects, even under circumstances of low-temperature stress. For instance, the tropical cockroach *Gromphadorhina coquereliana* exhibited a general decreasing trend in protein, carbohydrate, amino acid, and glycogen levels during the initial stage of cold stress, but glycogen levels became upregulated with fat metabolism during the later stage. Unlike solitary insects, honey bees do not enter hibernation during overwintering; instead, they form tight clusters, generate heat through the vibration of thoracic flight muscles in worker bees, and consume food reserves to maintain their minimum energy requirements. Therefore, there remains debate as to whether honey bees rely on fat metabolism to provide energy during overwintering. In this study, the downregulated DEGs of Hunchun bee in winter were significantly enriched in the fatty acid metabolism pathway. It was speculated that a slower metabolism rate of fatty acids could maintain a certain level of fatty acids in Hunchun bee [40]. On the one hand, these fatty acids can be converted into antifreeze protective agents to reduce cell damage caused by low temperatures. On the other hand, fatty acids are also an integral part of the ‘energy-saving model’ of honey bees during the overwintering period; that is, honey bees consume the sugar feed stored in the colony during the overwintering period to produce heat for overwintering [41]. However, in emergencies such as insufficient feed, the stored fatty acids in honey bees are slowly consumed to provide energy to ensure their survival during the long winter [42]. Of course, these speculations still need to be verified by further metabolic research.

### 4.4. Expression Regulation of Cold Resistance-Related Protein Genes

Besides substance metabolism, the synthesis of cold-resistant macromolecular proteins is also a major molecular mechanism of cold resistance in insects [43]. In the present study, we found that the shared upregulated DEGs in winter, as well as shared upregulated DEGs of the later overwintering intervals vs. the early overwintering period, were significantly enriched in the regulation of macromolecular biosynthesis processes. This suggests that overwintering honey bees also rely on the biosynthesis of cold-resistant macromolecular substances for cold resistance during the long winter season [44]. Gene annotation revealed that the shared DEGs of the overwintering period and of the mid- and late overwintering intervals included members of several candidate cold-resistant gene families, such as AFPs, HSPs, C_2_H_2_-ZFPs, STKs, and LRRCs. AFPs mainly inhibit the growth and recrystallization of ice crystals within solitary insects through adsorption to the ice crystal surface [45]. The significant upregulation of the AFP gene in the Hunchun bee within the late overwintering period suggests that this gene served an important role in inhibiting ice crystal formation in vivo during the overwintering process. HSPs can improve cell resistance to cryoprotectant toxicity and freeze–thaw damage, thereby enhancing cell survival [46]. In the present study, it was found that the expression levels of the HSP genes HSP60, HSP70, and HSP90 were lower in winter than in the summer breeding period. However, the three HSP genes showed fluctuating trends in the four overwintering intervals, suggesting that these genes play a protective role during overwintering in Hunchun bee. STKs are key enzymes involved in phosphorylation. They regulate downstream protein activity and participate in immune responses and nervous system functions through phosphorylation, thus influencing cell growth, metabolism, and stress responses [47]. C_2_H_2_-ZFPs are transcription factors that can regulate gene expression, and they serve an important role in the molecular cascade of anti-cold responses in honey bees by responding to temperature stress and regulating the expression of other cold resistance-related genes [48]. In the present study, 23 C_2_H_2_-ZFPs and 21 STKs were identified among the DEGs, of which 22 C_2_H_2_-ZFPs and 17 STKs were highly expressed during the overwintering period. This suggests that STKs and C_2_H_2_-ZFPs are the main cold-resistant functional genes in Hunchun bee. LRRCs are common candidate cold-resistant genes in plants, and they are evolutionarily conserved among many innate immunity-related proteins in plants and animals. They mainly mediate protein interactions and are involved in signaling, cell adhesion, and immune regulation [49]. In the present study, 12 LRRC family genes were identified among the DEGs, with 7 genes (including LRRC40, LRRC24, and LRRC15) exhibiting higher expression levels in the mid- and late overwintering period. This was consistent with the RT-qPCR results, indicating that the LRRC gene family is involved in the cold resistance mechanism of honey bees through the regulation of physiological processes such as signal transduction, cell adhesion, and immunoregulation.

### 4.5. Shared Genes of Hunchun Bee During Overwintering

In the present study, four shared genes of the overwintering period in Hunchun bee were identified by a combined analysis strategy involving a pairwise comparison of genes between overwintering intervals: sensory neuron membrane protein 1 (*SNMP1*), polypeptide N-acetylgalactosaminyltransferase, the nose resistant to fluoxetine protein, and ABC transporter G family member 20. Given the lack of research on the latter three genes in honey bees, the present study mainly focused on *SNMP1*. The SNMP1 protein is an important membrane protein for insect olfactory sensory neurons; it mediates pheromone receptor involvement in insect pheromone recognition and is highly expressed in pheromone receptor neurons of the trichoid sensilla. The gene directly interacts with DmelOR22a, and previous research has found that *SNMP1* in Lepidoptera is specifically expressed in the antennae of adult insects [50]. During overwintering in honey bees, the queen mandibular pheromone (QMP) secreted by the queen’s mandibular glands is an important chemical signal that maintains colony structure stability and behavioral coordination. Therefore, QMP serves a particularly critical role in colony behavior regulation and safe colony overwintering [51,52]. In the present study, SNMP1 was identified from the DEGs of worker bees over different overwintering intervals. It was deduced that the queen bee coordinated worker bee clustering and colony activities through QMP in the dark and quiet overwintering environment to improve the colony’s overwintering ability. We also identified two odor-binding proteins, OBP13 and OBP18, that are highly expressed in the overwintering period compared with the summer breeding periods, and three highly expressed odor-binding proteins, OBP4, OBP5, and OBP13, in the mid- and late overwintering intervals. These results suggest that the chemosensory system of the worker bees serves a regulatory function in sensing changes in the external environment and information exchange within the colony during the overwintering period, thereby ensuring successful survival over the cold season.

## 5. Conclusions

The present study revealed that overwintering in Hunchun bee is dependent on a variety of synergistic approaches. It was speculated that they attenuate winter-driven stress by maintaining osmotic pressure balance, reducing fatty acid metabolism, improving antioxidant capacity, and synthesizing cold-resistant macromolecular proteins during overwintering. Our results also suggested that chemical signal perception may serve a role in maintaining the stability of overwintering bee colonies. The key genes and pathways of cold resistance in Hunchun bee were systematically identified in this study. This study not only fills the gap in our understanding the molecular mechanism of *Apis mellifera* overwintering but also provides a theoretical basis for improving feeding and management during overwintering.

## Data Availability

The datasets presented in this study can be found in online repositories. The names of the repository/repositories and accession number(s) can be found below: NCBI SRA database (accession number: PRJNA1354183).

## Supplementary Materials

The following supporting information can be downloaded at https://www.mdpi.com/article/10.3390/insects17010059/s1, Figure S1: Volcano plot of genes differentially expressed between summer and winter; Table S1: The information of primers in RT-qPCR; Table S2: Quality analysis of sequencing data and comparison results of reference genomes; Table S3: Volcano plot data of genes differentially expressed between summer and winter; Table S4: Shared DEGs between four overwintering intervals and the summer breeding period; Table S5: Gene function enrichment analysis of the shared DEGs between four overwintering intervals and the summer breeding period.
