# Supplementary figures and images for: Transcriptomic Analysis of the Cold Resistance Mechanisms During Overwintering in Apis mellifera
Source: Insects. 2026 Jan 1;17(1):59. doi: 10.3390/insects17010059 (PMC12841635; doi:10.3390/insects17010059)

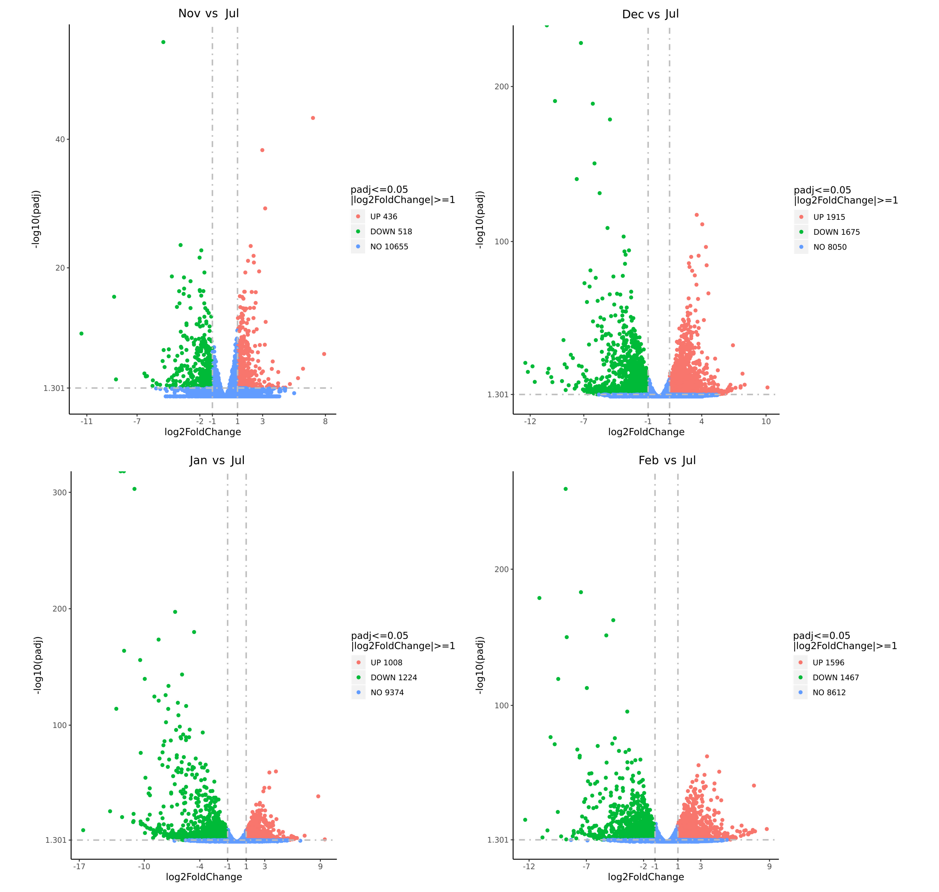

Supplement: Supplementary file 1 [file insects-17-00059-s001.zip › Figure S1. Volcano Plot of Genes Differentially Expressed Between Summer and Winter.png]
